# Evaluation of nanoparticle tracking analysis for total virus particle determination

**DOI:** 10.1186/1743-422X-9-265

**Published:** 2012-11-12

**Authors:** Petra Kramberger, Mateja Ciringer, Aleš Štrancar, Matjaž Peterka

**Affiliations:** 1 BIA Separations, d.o.o, Mirce 21, SI-5270, Ajdovščina, Slovenia; 2 CO-BIK, Velika pot 22, SI-5250, Solkan, Slovenia

**Keywords:** NanoSight, NTA, Adenovirus 5, Influenza, Latex particles, CIM monolithic column

## Abstract

The NanoSight LM10 with Nanoparticle tracking analysis (NTA) software was evaluated for the quantification of latex particles, adenovirus 5, and influenza virus. The inter-day variability was determined by measuring the same sample over several consecutive days and the method’s accuracy was demonstrated by using known concentrations of the subject particles. NTA analysis was also used to quantify chromatographic fractions of adenovirus and influenza virus after purification on a CIM monolithic column. NTA results were compared and evaluated against hemagglutination (HA) and end point dilution assay, determining total and infection virus particle number, respectively. The results demonstrated that nanoparticle tracking analysis is a method for fast estimation of virus concentration in different samples. In addition, it can provide a better insight into the sample status, regarding the level of virus aggregation.

## Introduction

Vaccines play an important role in the prevention and treatment of disease. Although vaccines have been used for decades, there has only recently been a concerted effort to optimize their production and improve vaccine safety and efficacy [1-3]. Apart from vaccines, where virus particles work as a preventive tool, they also have the potential to work as therapeutics: viral vectors for gene therapy [4-7] and bacteriophages for the treatment of bacterial infections [8-11].

The downstream processing (DSP) of vaccines represents a considerable production cost. During these purification steps, it is critical to monitor impurity removal and virus recovery in order to stop production runs as early as possible if a problem is detected. Since DSP strongly depends on the consistency of the up-stream process (USP), virus titre monitoring during USP is just as critical and important as during DSP.

The infective viral titre is determined by using the tissue culture infectious dose 50 (TCID50), plaque assay and end point dilution assay. In contrast to infectivity assays, which are laborious and time-consuming, total virus particles quantification methods are in general relatively quick, but have some other drawbacks. Spectrophotometric-based measurement of virus concentration (absorbance ratio 260/280 nm) can be applied only for highly purified virus preparations; quantitative real time polymerase chain reaction (qPCR) measures gene copy rather than particles and thus excludes empty capsids from the analysis [12]; transmission electron microscopy (TEM) has high variability in determining total virus counts and is quite time consuming and costly.

None of the above mentioned (infectivity and non-infectivity) methods enables the assessment of virus aggregation and complex formation. In a non-natural environment, such as low pH for influenza virus [13], viruses tend to aggregate with other virus particles or form complexes with impurities [14]. During DSP, these need to be removed as soon as possible to prevent or minimize further aggregation. At the later stages of DSP, aggregates occur due to high virus concentration [15,16].

The level of virus aggregation should be monitored throughout the downstream processing because this phenomenon can influence the behavior of some viruses like influenza A virus where aggregation inhibits virus hemagglutination activity and infectivity [17]. This consequently impacts the results obtained with virus quantification methods used at the end of DSP when vaccine dose is determined on the basis of infectivity or non-infectivity assays.

Factors that contribute to particle aggregation and methods to prevent its occurrence during virus purification and formulation were highlighted by Wright et al. [14]. Using recombinant virus vector AAV2, they demonstrated that aggregation cannot be prevented by different sugars or surfactants, but can be using various salts at concentrations corresponding to a solution ionic strengths of more than 200 mM [14].

For determination of virus particle size and distribution (aggregation), field flow fractionation connected with multiangle light scattering (FFF-MALS) and size exclusion chromatography coupled with MALS (SEC-MALS) were evaluated by Wei et al. [12]. These methods were than compared with several other methods: transmission electron microscopy (TEM), atomic force microscopy (AFM), quantitative reverse transcription polymerase chain reaction (RT Q-PCR), median tissue culture dose (TCID50), and the fluorescent focus assay (FFA). All of the methods evaluated have their own advantages and limitations and are complementary to each other. The selection of one specific method depends on the issue being examined [12].

For some applications, the ratio between total virus and infective virus particles is of great importance (e.g. gene therapy vectors), while for the others (e.g. inactivated viral vaccines) the total virus particle count is sufficient. To speed up downstream process development while simultaneously reducing costs, a total virus particle quantification method should be employed that is quick, reliable, and robust to enable the quantification of virus throughout the purification process.

Nanoparticle tracking analysis (NTA) is a system for sizing particles from about 30 to 1000 nm and ability to determine the concentration of particles in the solution within the concentration range from 10E+7 to 10E+9 particles/ ml depending on sample type. The technique combines laser light scattering microscopy with a charge-coupled device (CCD) camera operating at 30 frames per second, which enables the visualization and recording of nanoparticles in solution. The NTA software is then able to identify and track individual nanoparticles moving under Brownian motion and the results allow particle number concentration to be recovered. NTA was already evaluated for the measurement of nanoparticles [18], drug delivery nanoparticles and protein aggregates [19] and recently, ASTM has published a guideline on the use of NTA for Measurement of Particle Size Distribution of Nanomaterials in Suspension [20].

Our goal was to evaluate the potential of the Nanoparticle tracking analysis for virus particle quantification and assessment of the level of virus particles aggregation. A direct comparison with hemagglutination assay and indirect comparison with End-point Dilution Assay was made in order to reveal the advantages and drawbacks of a technique.

## Materials and methods

### Latex particles, viruses and virus sample preparation

Latex particles (120 nm in diameter; standard deviation: 21 nm) were purchased from Christine Grőpl (Tulln, Austria). Approximate particle concentration (1.05E+12 LP/ml of latex particles) was provided by manufacturer.

Adenoviruses are medium-sized (90–100 nm), non-enveloped, icosahedral viruses composed of a nucleocapsid and a double-stranded linear DNA genome. Adenovirus 5 (Ad5) was propagated in HEK293 cells and released from the cells by three cycles of freezing in a dry ice/ethanol bath and thawing in a 37°C water bath. After centrifugation at 6.500 x g for 10 min, the supernatant was treated with benzonase (Merck, Darmstadt, Germany) for 1h at 37°C and filtered using a 0.22 μm PES filter (TPP, Trasadingen, Switzerland). The benzonase treated and filtered virus harvest was purified on a strong anion exchanger, CIM QA monolithic column, as previously reported by Lah et al. [21].

Influenza virus (H1N1) was propagated in Vero cells, using serum free medium. The virus was harvested, benzonase treated, and concentrated by tangential flow filtration (Pelicon® XL Device, 300.000 NMWC, Millipore). The concentrated virus harvest was then filtered using a 0.45 μm Chromafil® cellulose acetate (CA) membrane (Macherey-Nagel, Düren, Germany) and diluted 1:1 (v/v) with 50 mM HEPES pH 7.5 (equilibration buffer used for chromatography). The virus was purified on a strong cation exchanger, CIM SO3 monolithic column, using the method developed and described by Peterka et al. [22].

### Nanoparticle tracking analysis (NTA)

NTA measurements were performed using a NanoSight LM10 instrument (NanoSight, Amesbury, UK), consisting of a conventional optical microscope, Marlin charged coupled device (CCD) camera, and a LM10 unit (sample unit) with a laser light source. LM10 is the first generation instrument from the NanoSight Company, which in the mean time has developed devices with additional features and upgraded software.

Following the manufacturer’s instructions, we serially diluted all samples with sterile water or DPBS to reach a particle concentration suitable for analysis with NTA (1.0E+8 to 2.5E+9 particles/ml). We prepared at least two different sample dilutions for each sample and analyzed each one twice. The samples were injected into the LM unit (approximately 300 μl) with a 1 ml sterile syringe. The capturing settings (shutter and gain) and analyzing settings were manually set according to the protocol suggested in the Technical note ”How to make Concentration Measurements using NanoSight Equipment” (Technical Note, NanoSight, last updated 17/06/09) and then optimized for a specific virus or the latex particles. The NanoSight LM10 recorded 60 second sample videos which were than analyzed with the Nanoparticle Tracking Analysis (NTA) 2.0 Analytical software release version build 0125.

### Hemagglutination assay

To estimate the total number of influenza virus particles, a standard hemagglutination assay (HA) using standardized concentration of avian red blood cells (0.5% RBC) as previously described by John Hierholzer et al. [23,24] was used.

A serial twofold dilution of the virus was prepared in U-bottomed 96 well microtiter plates containing 50 μl DPBS. Into each well, a 50 μl of 0.5% solution of chicken red blood cells was added and the plate was incubated for 60 minutes at room temperature. HA titres (HA/ml) were read as the reciprocal dilution of the last well showing hemagglutination.

### End point dilution assay

The infective virus titre of Ad 5 was determined by End-point Dilution (EPD) Assay. The assay is based on observing the cytopathic effect (CPE) of serially diluted adenovirus samples in HEK293 cells cultured in 96-well plates. CPE was examined 7–10 days after infection and the virus titre was calculated based on the dilution factors.

### Chromatography

A CIM® monolithic column (BIA Separations, Ljubljana, Slovenia) with a dimension of 12 mm x 3 mm i.d. and bed volume of 0.34 ml were used for purification of adenovirus and influenza virus. The column was attached to a Knauer (Berlin, Germany) gradient HPLC system, consisting of two K-500 pumps, a UV–VIS detector K-2500 set to 280 nm, and a data acquisition and control station with a PEEK capillary tube (i.d. 0.75 mm). A conductivity meter (GE Healthcare, Uppsala, Sweden) was added to the system. The adenovirus was purified on a strong anion exchanger (CIM QA monolithic column) using a 50 mM Tris containing 2 mM MgCl_2_ and 0.4 M NaCl, pH 8 equilibration buffer. The influenza virus was purified on a strong cation exchanger (CIM SO3 monolithic column) using a 50 mM HEPES equilibration buffer, pH 7.5. Both viruses were eluted from the column using high NaCl concentration in equilibration buffer.

## Results and discussion

### Quantitation of latex particles

The NTA method was first characterized by quantifying latex particles with a diameter of 120 nm at a concentration of 1.05E+12 LP/ml. These latex particles were used as a model nanoparticle since their size is similar to that of the two tested viruses (adenovirus and influenza). We assumed that the NTA method settings determined for the latex particles would be applicable for the viruses as well.

The settings selected for the video capturing of the latex particles were set according to the manufacturer’s instructions. The videos were analyzed under different conditions by changing brightness, blur, gain, and detection threshold. By comparing the concentration of latex particles in differently diluted samples, analyzed with different settings, we were able to determine which settings have a smaller or greater effect on the result (data not shown). Similar to the previously published findings by Filipe et al. [19], it was observed that the settings that have a major influence on the results are: shutter, gain, blur and detection threshold. Therefore, these settings were kept the same for the analysis of several dilutions of the same latex particle sample.

The videos were analyzed using the optimal settings determined for latex particle quantification with NTA: brightness −15, gain 2, blur 5x5 and detection threshold 30 (Table 1). Results obtained were subjected to the screening: The number of completed tracks had to be at least 200 or above and the average number of particles/frame had to be between 20 and 60. We also found that the ratio between the theoretical factor and a measured one which needed to be between 0.8 and 1.2 (Table 1, last column) was also important.

**Table 1:** Analysis of 120 nm latex particles

**Dilution**	**Measurement No.**	**Particles/frame**	**Completed tracks**	**Mean particle size (nm)**	**Measured conc.(LP/ml)**	**Average measured conc. (LP/ml)**	**Concentration (LP/ml)**	**Measured factor (MF)**	**Theoretical factor (TF)**	**TF/MF**
300	1	91*	2896	115	1,52E+09					
	2	79*	2407	119	1,27E+09	1,4E+09	4,2E+11			
500	1	120*	4179	100	1,93E+09			0,7	1,7	2,3
	2	115*	3999	103	1,86E+09	1,9E+09	9,5E+11			
900	1	87*	2621	125	1,40E+09			1,4	1,8	1,3
	2	80*	2253	133	1,29E+09	1,3E+09	1,2E+12			
1000	1	64*	2004	125	1,03E+09					
	2	35	755	143	5,69E+08					
	3	23	575	131	3,78E+08	4,7E+08	4,7E+11			
1500	1	61*	2005	106	9,92E+08			0,6	1,5	2,6
	2	52	1688	110	8,35E+08	8,4E+08	1,3E+12**			
2000	1	29	748	130	4,71E+08			1,7	1,3	0,8
	2	33	1004	109	5,38E+08	5,0E+08	1,0E+12**			
3000	1	13*	343	149	2,16E+08			1,3	1,5	1,2
	2	24	640	146	3,90E+08					
	3	24	681	127	3,94E+08	3,9E+08	1,2E+12**			
							1,15E+12	Final concentration (LP/ml)		

* Measurements where the “particle/frame” was higher than 60 or lower than 20 (consequently these measurements were eliminated from final calculation).

**Concentration values with TF/MF factor between 0.8 and 1.2 (these values were used to calculate the original concentration of latex particles).

**Figure 1:**
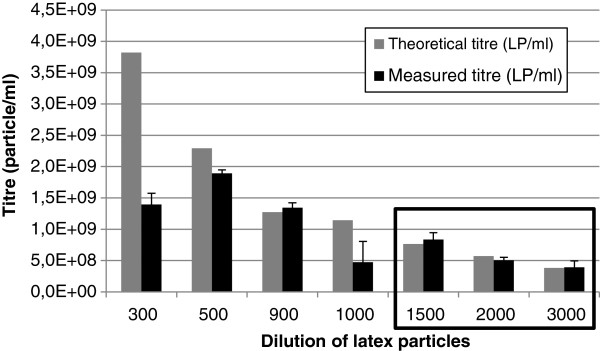
**Latex particle analysis: linearity of dilution.** Grey columns represent the theoretical titre of latex particles, while the black columns represent the measured titre. Only the last three dilutions measured fulfilled both criteria (average particle number in the range between 20 and 60 and the TF/MF factor between 0.8 and 1.2) and were used to calculate the original concentration of latex particles.

This means that we allowed up to a 20% deviation in the titre determined between the two consecutive dilutions of the same sample to recognize the measurement as a “relevant” one. The limit of 20% was set on the basis of recommendations from the U.S. Department of Health and Human Services, Food and Drug Administration, and others set in the guidance for Bioanalytical Method Validation [25]. By introducing this last criterion, an additional (back-up) system for the recognition of relevant measurements was established. Linearity-of-dilution is shown in Figure 1.

We also monitored the operator-to-operator variability (data not shown) and it was determined that it is within the acceptable range of 15-20% for the accuracy of bioanalytical methods [25].

### Quantitation of adenoviruses

Since the particle size of an adenovirus is 100 nm in diameter which is similar to the 120 nm latex particles, the parameters used for the analysis were similar to those of the latex particles. The video capture used a shutter speed of between 1340 and 1500 and the gain was set to the maximal value. The videos were processed with the brightness set to −15, gain 2, blur 3x3 and detection threshold 30.

To establish the inter-day variability/repeatability of the NTA method, the same adenovirus sample was assayed for 4 consecutive days. Between the measurements, the adenovirus sample was stored at −82°C. Each day the adenovirus sample was thawed, two dilutions were prepared (with DPBS), and each dilution was analyzed twice. The inter-day variability of the method was determined to be 14% (Table 2). We are presuming that the freeze-thawing of the sample did not have any effect (aggregation or disruption of virus particles) on the adenovirus total particles titre. This is reasonable since the average particle size during the course of experiment was not changed.

**Table 2:** Inter-day variability of the NTA method

**Sample**	**Dilution**	**Measured conc. (VP/ml)**	**Average measured conc (VP/ml)**	**Sample conc. (VP/ml)**	**MF**	**TF**	**TF/MF**	**Conc. (VP/ml)**
Day 1	20	6,90E+08						
	20	4,27E+08	5,59E+08	1,12E+10				
	30	4,63E+08			1,3	1,5	1,1	1,2E+10
	30	3,73E+08	4,18E+08	1,25E+10				
Day 2	20	6,08E+08						
	20	8,12E+08	7,10E+08	1,42E+10				
	30	4,89E+08			1,4	1,5	1,1	1,5E+10
	30	5,49E+08	5,19E+08	1,56E+10				
Day 3	20	6,28E+08						
	20	6,75E+08	6,52E+08	1,30E+10				
	30	5,50E+08			1,3	1,5	1,2	1,4E+10
	30	4,60E+08	5,05E+08	1,52E+10				
Day 4	20	5,73E+08						
	20	5,35E+08	5,54E+08	1,11E+10				
	30	3,79E+08			1,5	1,5	1,0	1,1E+10
	30	3,52E+08	3,66E+08	1,10E+10				
Average								1,3E+10
STDV								1,8E+09
%STDV								14,1

Inter-day variability of the NTA method determined by measuring the same sample (Ad5 virus) for 4 consecutive days. MF- the ratio between concentrations of two sample dilutions determined with NTA; TF-the ratio between two dilutions of the same sample analyzed.

NTA accuracy was determined using a spike-and-recovery method. The original latex particle (LP) solution, containing particles with a diameter of 120 nm at a concentration of 1.05E+12 LP/ml, was diluted 10-fold with DPBS. The resulting theoretical concentration was 1.05E+11 LP/ml which was then used as a spike.

**Figure 2:**
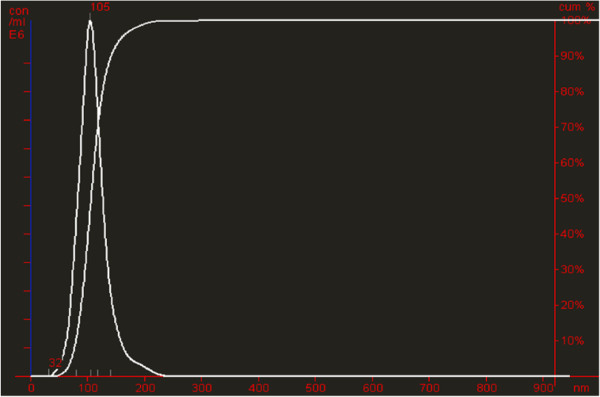
**Particle size distribution of latex spiking solution.** Particle size distribution of ten-fold (1:9, v/v) diluted original latex solution (spiking solution) added to DPBS (measured latex spiking solution).

Since 5 μl of the 10-fold diluted latex solution was added to the adenovirus sample as a spike, the total particle titre added corresponds to 5.25E+8 LP/5 μl. In Table 3, this is listed as the “theoretical latex spike”. In parallel, the actual titre of the ten-fold diluted latex solution was measured: 5 μl of the spiking solution was added to the DPBS buffer and analyzed with NTA (“measured latex spike”, Figure 2). The theoretical and measured latex spike concentrations was very similar (Table 3) and were used to determine the recovery of the latex spike added to the adenovirus samples.

**Table 3:** Accuracy of NTA determined by spike-and-recovery method

	**Adenovirus harvest**	**CIM QA purified adenovirus**
Dilution of Ad sample for NTA analysis	30	500
Theoretical latex spike (LP/5μl)	5,25E+08	5,25E+08
Measured latex spike (LP/5μl)	5,15E+08	5,42E+08
Concentration of non-spiked Ad sample (VP/ml)	3,43E+08	3,57E+08
Concentration of spiked Ad sample (particles/ml)	7,84E+08	8,27E+08
∆ (spiked Ad sample-non-spiked Ad sample)	4,42E+08	4,70E+08
Recovery of latex spike sample according to measured spike (%)	85,7	86,7
Recovery of latex spike sample according to theoretical spike (%)	84,1	89,5

Since the adenovirus particle size is relatively close to the latex particles, we used the same shutter speed and gain mode for the NT analysis of spiked and non-spiked samples.

Two adenovirus samples, differing in virus purity and concentration, were chosen for the analysis. The first adenovirus sample was treated with benzonase and filtered through a 0.22 μm filter and the second was purified using an anion exchange chromatography resin, CIM QA monolithic column. Both samples were analyzed with and without the addition of the spike and the concentrations obtained were compared and spike recovery was calculated (Table 3). Spike recovery was very similar for the non-purified and CIM purified adenovirus, although the virus concentrations in these two samples differed greatly. Since the theoretical (calculated) concentration of the latex spike sample differed from the measured one by only 3%, the recovery of the latex spike was also very close. According to spike recovery (around 85%), NTA underestimates the concentration of particles (latex and/or adenovirus) in the sample for around 15%. The accuracy of the measurements is still within the 20% as accepted by Bioanalytical Method Validation [25] however the measurements tend to show a small systematic shift for witch the reason is still unclear.

**Figure 3:**
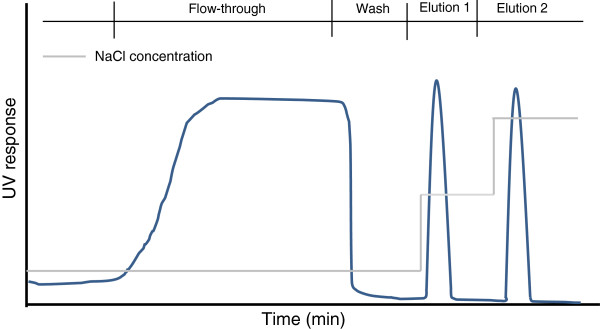
**Shematical chromatograph of virus purification.** Flow-through fraction (non- bound compounds), wash (weakly bound compounds washed from the column), elution 1 (the main virus fraction), elution 2 (elution of strongly bound impurities-usually host cell DNA). Selective elution in this case is achieved by increasing salt concentration (indicated with the grey line).

To minimize the inter-day and operator to operator variability of NTA, a set of chromatographic fractions (schematically shown on Figure 3) obtained during adenovirus purification on a CIM QA monolithic column were analyzed within the same day by the same operator. In addition, the fractions were also analyzed by End point dilution (EPD) assay.

Since EPD enables the quantification of the infective virus particles and NTA the quantification of total virus particles, the two methods cannot be directly compared. By indirectly comparing them using virus recovery (Table 4), there was a good correlation between both methods.

**Table 4:** Comparison of EPD and NTA method

		**EPD**				**NTA**	
**Fraction**	**Volume (ml)**	**PFU/ml**	**PFU total**	**PFU rec.(%)**	**VP/ml**	**VP total**	**VP rec. (%)**
Load	36	1,00E+08	3,60E+09		1,03E+10	3,71E+11	
Flow through 1	10,0	6,31E+02	6,31E+03	0,0	1,12E+08	1,12E+09	0,3
Flow through 2	26,0	1,00E+03	2,60E+04	0,0	1,59E+08	4,13E+09	1,1
Wash	23,0	NA			3,03E+08	6,97E+09	2
Elution 1	1,2	2,00E+09	2,40E+09	66,7	1,79E+11	2,14E+11	57,8
Elution 2	3,0	6,31E+06	1,89E+07	0,5	1,14E+09	3,41E+09	1
SUM				67,2			62,0

Analysis of adenovirus purification on CIM QA monolithic column by NTA and EPD assay.

The method used to purify the adenovirus was not optimized and only performed on a laboratory scale for the purpose of evaluating the NTA method. Therefore, the virus recovery as well as the ratio between infective and non-infective particles is not optimal.

### Quantitation of influenza virus

In order to compare the NTA method with another frequently used method for total virus quantification, two sets of samples (chromatographic fractions) obtained during influenza virus purification on a CIM monolithic column were analyzed by the NTA method and hemagglutination assay.

The hemagglutination assay is based on the binding (agglutination) of red blood cells (RBCs) which occurs after a certain concentration of viruses have attached to the RBCs surface. The ability of viruses to agglutinate RBCs is rarely linked to their infectivity [26] therefore the method can be applied to quantify the total number of virus particles. By serially diluting a virus suspension and adding a standard amount of RBCs, an estimation of the virus particles presence (in HA units/ml) was made and compared with the virus titre determined by the NTA method (Table 5).

**Table 5:** Comparison of HA and NTA method

		**HA**				**NTA**	
	**V (ml)**	**HA (HA/ml)**	**Total HA (HA)**	**Recovery (%)**	**NTA (VP/ml)**	**Total titer (VP)**	**Recovery (%)**
Load	1,5	128	192,0		5,7E+09	8,6E+09	
1 FT	2,9	<1			4,1E+08	1,2E+09	13,8
1 E1	1	128	128,0	66,7	5,8E+09	5,8E+09	68,0
1 E2	1,15	4	4,6	2,4	1,5E+08	1,7E+08	2,0
Total recovery (%)				69,1			83,0
2 FT	2,4	<1			6,0E+08	1,4E+09	16,7
2 E1	0,9	128	115,2	60,0	7,2E+09	6,5E+09	75,4
2 E2	1,1	4	4,4	2,3	3,7E+08	4,1E+08	4,7
Total recovery (%)				62,3			96,8

Analysis of chromatographic samples collected during influenza virus purification on two different CIM SO3 monolithic columns by HA and NTA method; FT (flow-through fraction) and E (elution fractions).

**Figure 4:**
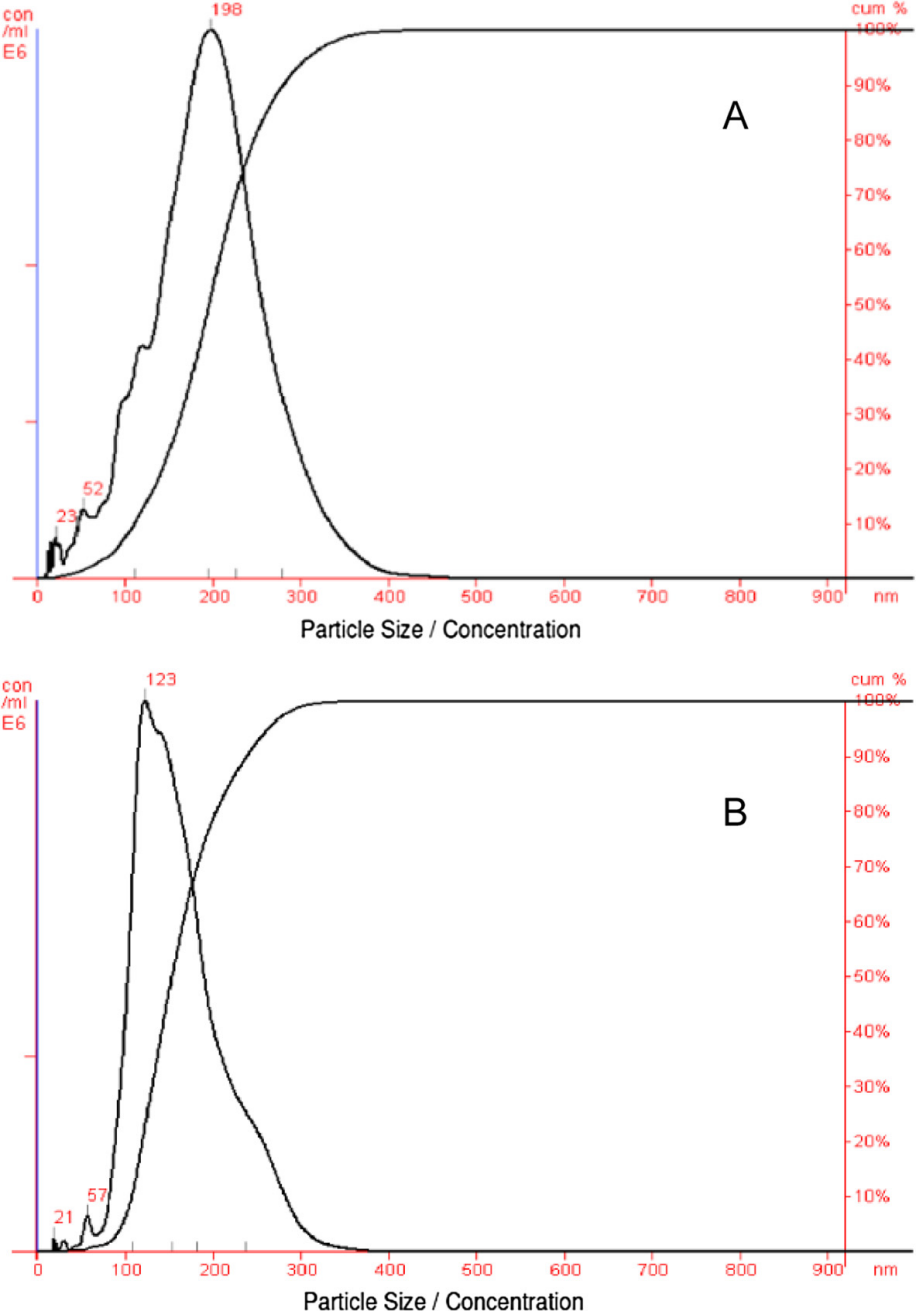
**Particle size distribution of influenza virus.** Particle size distribution of influenza virus before (**A**) and after (**B**) purification on CIM SO3 monolithic column; both samples diluted 1:20 (v/v) with DPBS.

In the majority of the samples tested, the virus presence (compared on the level of the virus recovery) obtained by the HA and NTA method were comparable. In 6 out of 7 samples analyzed, the NTA method determined a slightly higher virus content and consequently virus recovery compared to the HA assay. This was to some extent expected since the NTA sample analysis showed a broader size distribution of peaks than expected based upon the size of the influenza virus (Figure 4). This indicates that the samples analyzed contained to some extent virus particle aggregates. This is especially evident for the influenza virus sample before purification on CIM SO3 column (Figure 4A).

The main difference between the results obtained by both methods is the virus presence determined in the flow-through fraction (in both chromatographic purifications of influenza virus). While no virus (or less than 1 HA unit/ml) was detected with the HA assay, the NTA measurement showed the presence of the virus reflecting a virus recovery of 13% and 16%, respectively. This discrepancy between the measurements of the virus presence in the flow-through fraction resulted in a relatively big difference in total virus recovery determined by both methods.

There are two hypothesis why no virus could be determined in the flow-through fraction by the HA method, while this was not the case with the NTA method. The first one is connected to virus aggregation in the flow-through fraction resulting in an underestimation of virus particles determined with the HA assay. This phenomenon was previously described by Kalbfuss et al. [27]. But this theory could only be supported if the average size of the virus particles in the flow-through fraction would indicate aggregation, which was not the case. The second hypothesis is based on the fact that in the flow-through fraction the virus particles which the NTA method was able to detect lost the ability to agglutinate erythrocytes and could therefore not be detected by the HA assay. Since the ability of viruses to agglutinate RBCs is rarely linked to the virus infectivity [26], this hypothesis would be very hard to prove even with an infectivity assay.

In any case, the NTA method proved to be suitable for a quick estimation of total virus particles in chromatographic samples of influenza virus and could be used as a sole or a complementary method for virus recovery estimations.

In general, the data obtained by the NTA should be interpreted with care. It is very important that enough particles are tracked and that the right settings are chosen. After analysis only the “relevant” data should be used for the calculation of the final particle concentration, following the criteria discussed earlier (the number of completed tracks, the average number of particles/frame and the ratio between the theoretical factor and a measured one).

## Conclusions

During downstream processing development, it is very important to monitor virus recovery. To determine the virus recovery, different virus samples (e.g. chromatographic fractions) containing different virus titres have to be analyzed and compared with the starting material. Titres of different virus samples can only be compared if they are analyzed under the same conditions. In the case of NTA, this means the analysis is performed using the same parameters. To determine this, the optimal settings to enable virus quantification over a large span of virus titters should be identified.

Since the virus material during DSP development contains different concentration of viruses, linearity of the method is a key feature. The optimal set of NTA parameters should therefore enable virus quantification over a large span of virus titters. The method’s linearity can be determined by assaying different dilutions of the virus sample and calculating the ratio between the theoretical linearity factor and a measured one (TF/MF ratio).

Inter-day variability of the method should also be taken into account by comparing several samples on different days. In addition, the accuracy of the NTA method, shown by spike and recovery method indicates that the NTA method underestimates the titre of adenovirus for approximately 15%.

Comparison of NTA with HA assay showed that NTA can successfully substitute the HA method when infectivity of the virus is not an issue. In addition, it can provide a better insight into the sample status, regarding the level of virus aggregation.

## Competing interests

The authors work for the company BIA Separations (Ajdovščina, Slovenia) and/or for the Center of Excellence for Biosensors, lnstrumentation and Process Control (Solkan, Slovenia). None of the authors have any relation to the technology tested. We have never received reimbursements, fees, funding, or salary from an organization that may in any way gain or lose financially from the publication of this manuscript, either now or in the future. Such an organization does not finance this manuscript (article-processing charge). The authors do not hold any stocks or shares in an organization that may in any way gain or lose financially from the publication of this manuscript, either now or in the future. The authors do not hold or are currently applying for any patents relating to the content of the manuscript and none of the authors have received reimbursements, fees, funding, or salary from an organization that holds or has applied for patents relating to the content of the manuscript. There are none non-financial competing interests (political, personal, religious, academic, ideological, intellectual, commercial or any other) to declare in relation to this manuscript.

## Authors’ contributions

MP and PK designed and coordinated the study, MC and PK carried out the NTA measurements, PK participated in the chromatography part and drafted the manuscript. MP and AŠ have been involved in revising the manuscript critically and made substantial contributions to the interpretation and discussion of the results. All authors read and approved the final manuscript.
